# The effect of HIV prevention products on incentives to supply condomless commercial sex among female sex workers in South Africa

**DOI:** 10.1002/hec.3784

**Published:** 2018-06-21

**Authors:** Matthew Quaife, Peter Vickerman, Shanthi Manian, Robyn Eakle, Maria A. Cabrera‐Escobar, Sinead Delany‐Moretlwe, Fern Terris‐Prestholt

**Affiliations:** ^1^ Department of Global Health and Development London School of Hygiene and Tropical Medicine London UK; ^2^ Wits RHI University of the Witwatersrand Johannesburg South Africa; ^3^ School of Social and Community Medicine University of Bristol Bristol UK; ^4^ Washington State University Pullman Washington USA

**Keywords:** condom differential, economics of sex work, HIV prevention, risk compensation, South Africa

## Abstract

Evidence suggests that economic factors play an important role in commercial sex work, in particular that condomless sex commands a price premium relative to condom‐protected sex. This paper explores whether the use of a new HIV prevention product, with 100% efficacy but modeled after pre‐exposure prophylaxis (PrEP), could change the price and quantity of condomless commercial sex supplied. We collected stated preference data from 122 HIV‐negative female sex workers in urban South Africa, using a repeated choice experiment to simulate the impact of using PrEP on choices. Results suggest that the price premium for condomless sex would decrease by 73% with PrEP use and the quantity of condomless sex is predicted to increase by a factor of 2.27. Act price does not significantly affect choices without protection but strongly influences choices under full HIV protection. The utility offered by condoms reduces by around 15% under PrEP use. Because new HIV prevention products do not protect against other STIs or pregnancy, the unintended consequences of introducing HIV prevention products should be closely monitored, whereas users should not face stigma or blame for reacting rationally to exogenous changes to market conditions.

## 1 INTRODUCTION

Women engaged in commercial sex, or female sex workers (FSWs), face daily risks of HIV acquisition (Bekker et al., 2015), not least in urban South Africa where HIV prevalence among FSWs is estimated to be as high as 72% in some cities (UCSF, Anova Health Institute, & WRHI, 2015). FSWs face different choices and incentives from other high‐risk populations, because, in addition to navigating the complexities of sexual and personal risk in noncommercial sexual relationships, economic and social inequalities mean that FSWs often face strong competition from FSW colleagues, client resistance to condom use, and threats and use of violence from clients and police (Beattie et al., 2010; Deering et al., 2014; Pronyk et al., 2006; Wojcicki & Malala, 2001). A growing topic for economic research, analyses have sought to explain entry into and persistence of the industry (Cunningham & Kendall, 2014; Della Giusta, Di Tommaso, & Strom, 2009; Edlund & Korn, 2002), the intensive margin of risk/benefit trade‐offs (Gertler, Shah, & Bertozzi, 2005; Rao, Gupta, Lokshin, & Jana, 2003), and vulnerabilities faced by FSWs from broader economic forces (Cunningham, Kendall, & Lexecon, 2009; Robinson & Yeh, 2011).

Financial incentives in sex work may directly affect the HIV, STI, and pregnancy risk FSWs bear. For example, econometric work has estimated a price premium for condomless sex relative to protected sex, referred in the literature as the condom differential. Anecdotal evidence of a differential (e.g., Wojcicki & Malala, 2001) was first empirically estimated by Rao et al. (2003) who found that Indian FSWs who used condoms consistently faced income losses of up to 79%, whereas further econometric evidence has estimated price premia in different contexts between 7% (Belgium and the Netherlands) and 81% (Bangladesh; Arunachalam & Shah, 2013; de la Torre, Havenner, Adams, & Ng, 2010; Gertler et al., 2005; Levitt & Venkatesh, 2007; Muravyev & Talavera, 2013; Rao et al., 2003; Robinson & Yeh, 2011). In many circumstances, FSWs are highly dependent on sex work to support themselves and their families (Beattie et al., 2012) and therefore may be at heightened risk when offered more money for unprotected sex.

The HIV prevention landscape has evolved considerably in recent years, not least due to emerging evidence that antiretroviral (ARV) drugs can be used for HIV prevention. The HPTN 052 trial demonstrated the ability of ARVs to reduce the infectiousness of HIV‐positive persons through suppressing viral loads (Cohen et al., 2011) and has led to the development of pre‐exposure prophylaxis (PrEP), which has shown to offer a high degree of protection when used by HIV‐negative persons in different contexts and populations worldwide (Fonner et al., 2016). In 2012, the World Health Organization recommended oral PrEP to be used in groups at high risk of HIV acquisition (World Health Organization, 2015). By implementing national guidelines for the provision of PrEP among FSWs in 2016, South Africa became one of the first countries worldwide to offer ARV‐based prevention to any group (Department of Health South Africa, 2016). In such early stages of PrEP distribution, it is critical that we fully understand the impact that PrEP might have on potential users, specifically to understand potential unintended consequences associated with sustained PrEP use which, to date, clinical trials have not considered.

We hypothesize that the introduction of a fully effective HIV prevention product will affect both the supply and demand of protected and condomless sex because an HIV‐negative user will bear less risk from engaging in condomless sex. Henceforth, when we refer to PrEP in this paper, we define it as a fully effective HIV prevention product. Using the theory of compensating differentials, we show that this would result in both (a) an increase in the quantity of condomless sex supplied and (b) a decrease in the price premium for condomless sex, under reasonable assumptions. To test this empirically, we interviewed 203 FSWs in Ekurhuleni Municipality, in the urban periphery of Johannesburg, South Africa.

An ideal study design to test this hypothesis would be to randomly allocate a group of FSWs to receive an actual PrEP product, with associated efficacy and attributions, and a group to receive nothing, comparing changes in pricing and the supply of protected and unprotected acts. However, this randomization was not practically or ethically possible, largely because of PrEP's proven efficacy, and we were not able to access the small number of FSWs using PrEP in South Africa when this study was conducted; we therefore take a different approach.

We used stated preference methods, a repeated discrete choice experiment (DCE) presented with and without framing of HIV protection, to estimate the effect of hypothetical PrEP use on FSW preferences for condom, price, and client characteristics. In addition to overcoming the issues above, this within‐participant design also avoids issues of potential endogeneity from selection into a PrEP program. Stated preference methods are used frequently in health (de Bekker‐Grob, Ryan, & Gerard, 2012), transport (Hess, Adler, & Polak, 2007; Li & Hensher, 2012), and environmental economics (Whittington & Pagiola, 2012), offering a flexible and theoretically robust approach to eliciting preferences towards products and situations that do not yet exist (Hensher, Rose, & Greene, 2015). Although their hypothetical nature means that stated and revealed preferences may not be perfectly concordant, empirical research in health has shown relatively small levels of hypothetical bias and reasonable external validity (Lancsar & Swait, 2014; Quaife, Terris‐Prestholt, di Tanna, & Vickerman, 2018; Telser & Zweifel, 2007). From the DCE, we find that PrEP use will reduce the price premium for condomless sex by 73%, and simulations indicate that the quantity of condomless sex supplied will more than double. When asked to respond as if using a 100% effective HIV prevention product, the utility provided by condom use was diminished by 15%. We find that FSWs do not make choices based on price at present; however, the price paid by a client would strongly influences choice under full HIV protection.

This paper makes two contributions to the economic literature on sex work and HIV prevention and provides new insights into a critical population in the country with the world's largest HIV burden. First, to date, a body of economic work exists that explores the nature and challenges of sex work, yet no research has looked at the potential impact of new PrEP products on the incentives, risks, and pressures that FSWs face. Second, despite a high estimated prevalence among a national population of 130,000 FSWs (SWEAT, 2015), to date, this is the only published piece of economic work among South African FSWs.

## 2 THEORETICAL MODEL OF COMMERCIAL SEX

The following model is an extension of that devised by Gertler et al. (2005), the first paper to formalize the FSW/client relationship in a partial equilibrium framework. First, we describe their model, which considers the market where condoms are the only way of preventing HIV transmission, before extending it to consider the effect of a new prevention product.

### 2.1 FSW and client utility payoffs

Take a client's willingness to pay (utility) for sex to be V, whereas his maximum willingness to pay to not use a condom (disutility) is *β*. Then

$$Y_{\text{condom}}^{\text{client}}=V-\beta -P^{c},$$

where $Y_{\text{condom}}^{\text{client}}$ is a client's utility payoff from condom‐protected sex with a FSW and *P*^*c*^ is the price he pays her for protected sex. Considering his utility payoff from unprotected sex, the *β* element is dropped, and his payoff function becomes

$$Y_{\text{nocondom}}^{\text{client}}=V-P^{\mathrm{nc}},$$

where $Y_{\text{nocondom}}^{\text{client}}$ is a client's utility payoff from unprotected sex with a FSW and *P*^*nc*^ is the price he pays her for unprotected sex.

The utility payoffs for FSWs are

$$Y_{\text{condom}}^{\mathrm{FSW}}=P^{c}-W$$

and

$$Y_{\text{nocondom}}^{\mathrm{FSW}}=P^{\mathrm{nc}}-\gamma -W.$$

Thus, the FSW gains utility from the amount a client pays. However, when she engages in unprotected sex, she suffers a disutility from exposing herself to risk, discomfort, or other negative attributes of unprotected sex. Furthermore, the sex worker can expect to receive W from the next‐best use of her time.

We assume that clients and FSWs have a choice whether to engage in any type of sex, though allow for differences in bargaining power between the two groups. The model suggests that a FSW will supply unprotected sex if both she and the client agree not to use a condom. For FSWs, this will occur when the marginal revenue she receives from unprotected sex (*P*^*nc*^ − *P*^*c*^) is greater than or equal to her disutility from not using a condom (*γ*). For clients, this will occur when his marginal cost of not using a condom (*P*^*nc*^ − *P*^*c*^) is less than his disutility from condom use (*β*). Therefore, two conditions must hold for unprotected sex to occur:

β>γ

and

$$V>\left(W+\gamma \right).$$

In other words, that the maximum a client is willing to pay not to use a condom is greater than the maximum a FSW is willing to accept to take the risk and that the client's maximum willingness to pay for sex is greater than FSW's costs associated in noncondom sex.

We consider the effect of PrEP on client and FSW payoffs. First, we assume that the FSW's disutility from supplying unprotected sex declines. We define a new parameter, *η*, that represents this change so that her payoff from noncondom use is now

$$Y_{\text{nocondom}}^{\mathrm{FSW}}=P^{\mathrm{nc}}-\left(\gamma -\eta \right)-W,$$

where *η* is assumed to be positive. Similarly, we assume that male clients' utility from noncondom sex increases by the extent to which a client values HIV protection (which is now provided by PrEP). We represent the clients' utility gain from PrEP by *ξ* so that his payoff from noncondom use is now

$$Y_{\text{nocondom}}^{\text{client}}=V+\xi -P^{\mathrm{nc}},$$

where, again, *ξ* is assumed to be positive. Payoffs from condom use have not changed, so we retain the equilibrium price of unprotected sex from Gertler et al. (2005):

$$P^{C}=\left(1-\alpha \right)\left(V-\beta \right)+\mathrm{\alpha W}.$$

To find the new equilibrium price for noncondom use with PrEP, we maximize (*V* + *ξ* − *P*^*nc*^)^*α*^(*P*^*nc*^ − (*γ* − *η*) − *W*)^(1 − *α*)^ where *α* represents the client's bargaining power and (1‐ *α*) FSW bargaining power. We obtain

$$P^{\mathrm{nc}}=\left(1-\alpha \right)\left(V+\xi \right)+\alpha \left(\gamma -\eta +W\right).$$

Then, by subtracting *P*^*nc*^ − *P*^*c*^, we obtain the price differential between protected and unprotected sex:

$$P^{\mathrm{nc}}-P^{c}=\left(1-\alpha \right)\left(\beta +\xi \right)+\alpha \left(\gamma -\eta \right).$$

We can then compare the price premium for noncondom use with PrEP to that without PrEP:

$$\mathrm{Pre}m^{\text{PrEP}}-\mathrm{Pre}m^{\text{Orig}}=\left(1-\alpha \right)\xi -\alpha \eta .$$

The impact of PrEP on the price premium for noncondom use therefore depends on the relative bargaining weights of the FSW and client and the amount of utility the client and the sex worker each gain through the use of PrEP. The premium will decline if the following condition holds

(a) $$\frac{1-\alpha }{\alpha }<\frac{\eta }{\xi }.$$

Note that because both *η* and *ξ* are assumed positive, the following pair of assumptions generate a decline in the price premium under PrEP:

- The client's bargaining power is greater than the FSW's: *α* > 1 − *α*.
- The FSW gains more utility from PrEP than the client: *η* > ξ.

Under these two assumptions, which we believe are reasonable, the left hand side of Condition (a) is less than 1, whereas the right hand side is greater than 1, so the inequality is always satisfied.

## 3 EMPIRICAL METHODS

### 3.1 Study overview

The DCE was designed to elicit FSW preferences towards clients and sex act characteristics. To estimate the impact of PrEP on choices, the same 10 DCE tasks were presented twice to HIV‐negative FSWs, firstly with no framing: “You have the choice between providing services to one of two clients. Which would you prefer?” prior to a comprehensive description of a range of potential PrEP products to respondents. Then, the choice tasks were presented again with a protected framing: “Now I would like you to choose between 10 more sets of clients, but this time I would like you to make your choices imagining you were using a product which prevented you from getting infected with HIV. This means that there would be no risk of getting HIV from any client, whether or not you use a condom. Which would you prefer?”

Because respondents answered the same choice tasks twice (with different frames), we were able to test whether different choice task framings had a causal impact on FSW choices. Interviewers were experienced in sexual history surveying and were thoroughly trained and tested on their explanation of products and the DCE. Example products (an oral tablet, an injectable, a microbicide gel, and a intravaginal ring) were given to respondents to examine in an effort to imagine real‐life use, and interviewers answered any questions they had. In an additional DCE, preferences for product characteristics were consistent with prior expectations, demonstrating participant understanding of the protective benefits of products (Quaife et al., 2017; Quaife et al., 2016).

A respondent‐driven sampling (RDS) method (Johnston & Sabin, 2010) was used to recruit 203 FSWs in Ekurhuleni Municipality, Gauteng Province. Peer educators were used to locate sex work hotspots, and 12 were asked to act as seeds to start RDS chains in different areas (i.e., women working in brothels, hotels, or on the street). Seeds were invited to complete the survey and received ZAR 50 (USD $3.50) compensation for their time before they were given four coupons containing study information to distribute to other FSWs. When each referred FSW attended for an interview, their peer recruiter received a small incentive in the form of a ZAR 20 (USD $1.40) voucher.

The study was reviewed and approved by the University of the Witwatersrand Human Research Ethics Committee and the Research Ethics Committee at the London School of Hygiene and Tropical Medicine. All participation in the DCE, alongside supporting qualitative studies, was voluntary and subject to completion of written informed consent. The background survey asked several questions that led to a number of disclosures of distressing events, and a comprehensive distress protocol ensured that participants who disclosed these were referred to named persons at local clinics and nongovernmental organizations.

### 3.2 Questionnaire

#### 3.2.1 Selection of attributes and levels

The development of the DCE tasks was primarily based on thematic analysis of four focus group discussions. These were carried out with 52 self‐reported active FSW participants recruited through convenience sampling, with the assistance of FSW peer‐educators through a local nongovernmental organization. Qualitative analysis (presented elsewhere [Eakle et al., 2016]) generated a long list of potential DCE attributes, supplemented by key themes emerging from a scoping literature review. Final attributes and pictorial representations of choice tasks were then chosen through discussions with FSWs, peer educators, the FSW community advisory board at the Wits Reproductive Health and HIV Institute, and input from UK and South African experts in DCE methods.

**Figure 1:**
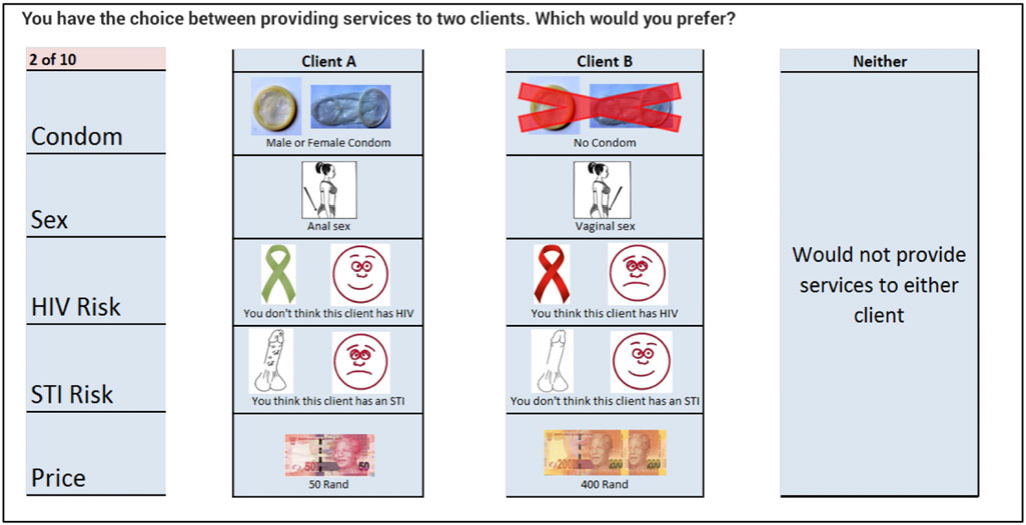
Example choice task without pre‐exposure prophylaxis framing [Colour figure can be viewed at http://wileyonlinelibrary.com]

Table 1 presents the final list of attributes and their levels, alongside the hypothesized direction of impact for each (positive or negative impact on utility). For example, we expect act price to have a positive coefficient and increase utility, whereas providing services to clients perceived to have an STI or HIV is likely to have a negative coefficient as it reduces utility. Figure 1 gives an example of a choice task as presented to respondents. The rest of the questionnaire captured data on a range of factors that may influence risk and pricing decisions such as socioeconomic characteristics, commercial and noncommercial sexual history, and exposure to structural factors such as intimate partner violence; these are used to explore preference heterogeneity though interaction terms.

**Table 1:** Attributes and levels

Attribute	Levels	Hypothesized coefficient sign
Price for sex	R100 (US $7.03), R200 (US $14.06), R400 (US $28.12), R800 (US $56.24)	+
Condom use	Condom, no condom	+, −
Type of sex	Vaginal sex, anal sex	+, −
Perceived client HIV risk	“You think this client has HIV,” “You don't think this client has HIV”	−, +
Perceived client STI risk	“You think this client has an STI,” “You don't think this client has an STI”	−, +
Task frame	No framing: “You have the choice between providing services to one of two clients. Which would you prefer?” PrEP framing: “Now I would like you to choose between 10 more sets of clients, but this time I would like you to make your choices imagining you were using a product which prevented you from getting infected with HIV. This means that there would be no risk of getting HIV from any client, whether or not you use a condom. Which would you prefer?”	

*Note*. PrEP: pre‐exposure prophylaxis.

#### 3.2.2 Experimental design

We apply best‐practice DCE design methods and use a fractional factorial design to generate the experimental design used in piloting, with pilot data subsequently providing Bayesian priors for the final design (Hensher et al., 2015). A 10‐task, D‐efficient unlabeled design was generated using NGENE software (ChoiceMetrics, 2012) with four unconstrained binary attributes (condom, sex, HIV risk, and STI risk) and one continuous linear attribute (price). Discussion continues in the literature on the best way to create efficient DCE designs, for example, however, D‐efficient designs are increasingly popular due to computational efficiency and statistical performance (Bliemer & Rose, 2011; de Bekker‐Grob et al., 2012; Johnson et al., 2013; Louviere, Hensher, & Swait, 2000).

### 3.3 Analysis

#### 3.3.1 Multinomial logit (MNL) estimation

Random utility models are used extensively in choice modeling, with the majority of health applications based on the MNL model (de Bekker‐Grob et al., 2012; McFadden, 1974). The MNL is computationally easy to implement, however, requires stringent assumptions including the absence of taste heterogeneity across respondents alongside restrictive patterns of substitution across alternatives. Nevertheless, the MNL is a useful model with which to explore choice patterns in the data before moving to more advanced estimation methods (Hensher et al., 2015).

Thus, the utility of respondent *n* for alternative *i* is given by a deterministic and measurable element *V*_*n*, *i*_ and a stochastic, unobserved element *ε*_*n*,*i*_:

(1) $$U_{n,i}=V_{n,i}+\varepsilon_{n,i}.$$

Assuming that *ε*_*n*, *i*_ has a type I extreme value distribution with values independently and identically distributed, the probability of choosing alternative *i* from choice set *j* is given by

(2) $$P_{n,i}=\frac{e^{V_{n,i}}}{\sum_{j=1}^{J}e^{V_{n,i}}}.$$

The MNL model is estimated by defining *V*_*n*, *i*_ as a vector of explanatory variables from the DCE design $X_{n,i}^{\prime }\beta_{i}$ and maximizing a log‐likelihood function in relation to *β*.

#### 3.3.2 Mixed multinomial logit (MMNL) estimation

As described by Hess, Bierlaire, and Polak (2005), we estimate a MMNL where the parameter vector *β* is assumed to be randomly distributed rather than fixed, such that *β*~*f*(*β*, Ω):

(3) $$P_{n,i}=\int_{\beta }P_{n,i}\left(\beta ,x_{n,i}\right)f\left(\beta ,\Omega \right)\mathrm{d\beta},$$

where Ω is a parameter vector of the distribution of the elements contained in *β*.

A restriction of the MMNL model is that the analyst needs to specify which parameters are randomly distributed across agents, as well as the way they are distributed (i.e., according to a normal, lognormal, or uniform distribution). The requirement of these assumptions is generally seen as a small cost for the ability of MMNL specifications to allow for taste heterogeneity, where preferences are allowed to vary across individuals (Hensher et al., 2015; Hess et al., 2005).

To test the effect of the DCE framing, we define *β*_*n*, *i*_ to include

(4) $$\beta_{n,i}=\alpha_{n}+\delta_{n}*\mathrm{PrE}{P_{\text{Frame}}}_{n},$$

where *α*_*n*_ is a coefficient vector capturing preferences with no framing, *PrEP*_*Frame* is a binary variable equal to 1 when the PrEP framed DCE is being presented, and *δ*_*n*_ is a coefficient vector capturing deviations between the two framings. We calculate robust t‐ratios to test the divergence of *δ* elements from zero. All parameters are estimated as random and normally distributed in MMNL estimation to reflect uncertainty in the distribution of their variance, and 1,000 Halton draws are created to estimate the model.

#### 3.3.3 Willingness to accept condomless sex

Analogous to willingness to pay estimates in the literature, we compute the relative importance of sex‐act attributes in monetary terms. For example, assuming a utility function which is linear in parameters, the willingness to accept condomless sex can be expressed as the monetary value:

(5) $$w_{k}=\frac{\beta_{k}}{\beta_{c}},$$

where *β*_*k*_ is the parameter for condom use and *β*_*c*_ is the price parameter. Because *β*_*k*_ and *β*_*c*_ are estimated with uncertainty, we consider the extent to which *w*_*k*_ is uncertain using the Delta method to estimate the standard error of *w*_*k*_. Assuming that $\hat{\beta }$ is asymptotically distributed such that $\hat{\beta_{k}}\overset{D}{\to }N\left(\beta ,\Omega_{\beta }\right)$, then, as shown in Bliemer and Rose (2013), the asymptotic standard error of *w*_*k*_ is

(6) $$\mathrm{se}\hat{\left(w_{k}\right)}=\frac{1}{\beta_{c}}\sqrt{\mathrm{var}\left(\beta_{k}\right)-2w_{k}\mathrm{cov}\left(\beta_{k},\beta_{c}\right)+w_{k}^{2}\mathrm{var}\left(\beta_{c}\right)}.$$

We use the Delta method to assess the significance of the ratio of condom use and price parameters (the condom differential) in the unframed DCE, the PrEP framed DCE, and to test whether the ratio *z*_*k*_ is significantly different from zero:

(7) $$z_{k}=\frac{\beta_{k}^{\text{prep}}/\beta_{c}^{\text{prep}}}{\beta_{k}^{\text{noprep}}/\beta_{c}^{\text{noprep}}}.$$

### 3.4 Simulations of behavior change

We apply predicted probability analysis to simulate the impact of PrEP on the price and supply of protected and condomless sex. As elsewhere in the health literature (Hensher et al., 2015; Philips et al., 2012; Sadique et al., 2013), we predict the supply response to PrEP introduction by summing the model coefficients with imputed attribute levels. As best practice in willingness to pay (WTP) studies from DCE data, we rescale results for simulation using revealed preference data. We take the absolute price premium as self‐reported by FSWs and reduce it by the factor *z*_*k*_ to generate the new price of condomless sex. We hold all attributes except condom use and price constant across framings and use MNL model outputs for transparency in simulations, simply substituting *V*_*n*, *i*_ into Equation (2).

## 4 RESULTS

### 4.1 Characteristics of respondents

Table 2 displays the descriptive statistics from the sample. In total, 203 FSWs were interviewed, of whom 81 (40%) self‐reported as being HIV positive. The remaining 122 HIV‐negative respondents completed both DCEs (thus acting as their own counterfactual), and we restrict analysis to this group. Each DCE had 10 choice tasks, meaning that we had 20 observations per respondent (10 without PrEP and 10 with PrEP) resulting in 2,440 choice data points. The average age of HIV‐negative respondents (29.48) was significantly (*p* > 0.01) lower than HIV‐positive respondents (32.95), and they were significantly less likely to have children (*p* > 0.01). In addition, HIV‐negative FSWs were more likely to make all their income from sex work (*p* > 0.01) and to have earned more from sex work in the last week (*p* = 0.05). The marriage rate was noticeably low in this sample at 2% for both HIV‐positive and ‐negative respondents, though 65% reported being in a relationship. Whilst reported rates of consistent condom use with commercial partners are much higher than other FSW surveys in South Africa (UCSF et al., 2015). Interestingly, HIV‐positive FSWs report charging significantly more than HIV‐negative FSWs (*p* = 0.04) for protected sex, whereas there is no significant difference for condomless acts. The price premium for unprotected sex among HIV‐negative FSWs is 1.8 times greater than that of HIV‐positive sex workers, though this difference is not statistically significant (*p* = 0.14).

**Table 2:** Descriptive statistics

Variable	Whole sample	%/(*SD*)	HIV negative (*n* = 122)	%/(*SD*)	HIV positive (*n* = 81)	%/(*SD*)	Difference between HIV positive and negative: *t*‐test *p* value
Age	30.87	(6.17)	29.48	(5.72)	32.95	(6.27)	>0.01
Secondary education	155	76	96.38	79	59.13	73	0.44
Married	4.06	2	2.44	2	1.62	2	0.59
In a relationship	132	65	55	63	77	68	0.49
Any children	182	90	104.92	86	76.95	95	>0.01
Always use condoms with clients	196	97	119	98	76.95	95	0.35
Experienced IPV in the last year	70	34	41.48	34	29.16	36	0.75
Used alcohol at last sex	32	16	18	15	14	17	0.15
Low household income (<R5,000/month)	125.86	62	69.54	57	55.89	69	0.1
Knows other FSWs engaging in condomless sex	81.2	40	45.14	37	35.64	44	0.28
In debt	78	38	42.7	35	34.83	43	0.26
All income made from sex work	174	86	108	89	65.61	81	>0.01
Amount charged to last client (ZAR)	103.57	(156.70)	114.75	(188.28)	86.73	(88.92)	0.21
Money earnt from sex work in last week (ZAR)	1,606.45	(1,392.34)	1,762.54	(1,548.29)	1,371.36	(1,084.49)	0.05
Average price charged for protected sex	83.06	(90.24)	72.38	(72.98)	99.14	(109.88)	0.04
Average price charged for condomless sex	411.16	(355.97)	466.52	(385.13)	347.5	(316.84)	0.28

*Note*. FSWs: female sex workers.

### 4.2 DCE results

Tables 3 and 4 show results from MNL and MMNL main effects estimation, respectively. Both tables show results for the unframed (Models 3A and 4A) and PrEP framed (Models 3B and 4B) DCEs. These tables also show the interaction specification of Equation (4) (Models 3C and 4C), where interaction coefficients represent the difference in preference weights between framings and where a statistically significant parameter indicates that choices under assumed 100% HIV protection differ from those under no protection.

**Table 3:** DCE results—Main effects MNL

	Model 3A: No frame MNL		Model 3B: PrEP framed MNL		Model 3C: Interacted MNL	
	Coeff.	*SE*	Coeff.	*SE*	Coeff.	*SE*
Price	0.0004	0.0005	0.0012***	0.0004	0.0004	0.0005
Condom use						
No condom						
Condom	4.53***	0.24	3.69***	0.22	4.53***	0.24
Type of sex						
Vaginal						
Anal	−2.89***	0.18	−2.98***	0.17	−2.89***	0.18
Perceived client HIV risk						
Do not think client has HIV						
Think client has HIV	−0.48***	0.14	−0.24**	0.13	−0.48***	0.14
Perceived client STI risk						
Do not think client has STI						
Think client has STI	−0.83***	0.16	−0.64***	0.15	−0.83***	0.16
Opt‐out (no services to either)	1.46***	0.17	1.00***	0.14	1.46***	0.17
Interactions						
PrEP framing × price					0.0008***	0.0006
PrEP framing × condom use					−0.84**	0.33
PrEP framing × anal sex					−0.09	0.25
PrEP framing × client HIV					0.24**	0.20
PrEP framing × client STI					0.19	0.22
PrEP framing × opt‐out					−0.46**	0.22
Model diagnostics						
Log likelihood	−587.31		−701.79		−1,289.10	
AIC	1,186.62		1,415.58		2,602.21	
BIC	1,216.60		1,445.58		2,670.51	
*N*	122		122		244	

*Note*. DCE: discrete choice experiment; MNL: multinomial logit; PrEP: pre‐exposure prophylaxis; AIC: Akaike information criteria; BIC: Bayesian information criteria.

*** Significant at 1%

** significant at 5%

* significant at 10%.

**Table 4:** DCE results—Main effects MMNL

	Model 4A: No framing MMNL		Model 4B: PrEP framed MMNL		Model 4C: Interacted MMNL	
	Coeff.	*SE*	Coeff.	*SE*	Coeff.	*SE*
Price	0.0002	0.0009	0.003**	0.0009	0.0018***	0.0006
Condom use						
No condom						
Condom	7.46***	0.56	6.34***	0.45	6.09***	0.33
Type of sex						
Vaginal						
Anal	−1.54***	0.33	−1.29***	0.33	−1.66**	0.29
Perceived client HIV risk						
Do not think client has HIV						
Think client has HIV	−0.89***	0.32	−0.97***	0.31	−0.69	0.21
Perceived client STI risk						
Do not think client has STI						
Think client has STI	−2.33***	0.41	−1.44***	0.30	−1.05***	0.21
Opt‐out (no services)	−1.58**	0.46	−1.67***	0.40	−0.92**	0.25
Interactions						
PrEP framing × price					0.0019**	0.0007
PrEP framing × condom use					−0.86**	0.42
PrEP framing × anal sex					−0.10	0.35
PrEP framing × client HIV					0.32	0.25
PrEP framing × client STI					0.33	0.28
PrEP framing × opt‐out					−0.11	0.30
Distribution parameters						
Price	0.0008***	0.0003	−0.0005	0.0005	−0.0006**	0.0002
Condom	0.16	0.17	−0.13	0.12	0.12	0.07
Anal	−4.00***	0.50	−4.85***	0.61	−2.08***	0.23
Think client has HIV	0.01	0.21	0.40*	0.20	0.09	0.13
Think client has STI	1.08***	0.25	0.51**	0.21	0.01	0.12
Opt‐out	3.11***	0.37	2.58***	0.25	2.34***	0.16
PrEP framing × price					0.0019**	0.0007
PrEP framing × condom use					−0.86	0.42
PrEP framing × anal sex					−0.10	0.35
PrEP framing × client HIV					0.32	0.25
PrEP framing × client STI					0.33	0.28
PrEP framing × opt‐out					−0.73***	0.15
Model diagnostics						
Log likelihood	−456.76		−538.05		−1,058.664	
AIC	937.5		1,100.1		2,165.33	
BIC	997.4		1,160.1		2,301.93	
*N*	122		122		244	

*Note*. DCE: discrete choice experiment; MMNL: mixed multinomial logit; PrEP: pre‐exposure prophylaxis; AIC: Akaike information criteria; BIC: Bayesian information criteria.

*** Significant at 1%

** significant at 5%

* significant at 10%.

Results are broadly consistent across MNL and MMNL specifications, and coefficient signs are in line with the theoretical expectations in Table 1. The nonsignificant price parameters in the unframed Models 3A and 4A suggest that FSWs do not choose clients based on price in current practice, but condom use, client characteristics, and the type of sex are all significantly important factors. However, the significant price parameter in Models 3B and 4B suggests that price will strongly influence choices after the introduction of PrEP. We test the hypothesis that price will become more important with PrEP use by examining the price * PrEP framing parameter in Models 3C and 4C, which show significance at the 95% level and therefore suggests that there is strong evidence for an increased influence of price on choices after the introduction of PrEP. Additionally, we find that the utility of condom‐protected sex is significantly reduced by PrEP use. In the MNL model, we find evidence that the framing of tasks was broadly understood due to the significant reduction in disutility from a client suspected as being HIV positive. However, this is not observed in the MMNL specification suggesting heterogeneity in the effect of framing across respondents. Both models suggest that, whereas FSWs do not favor clients who are perceived to have a STI, PrEP does not have a statistically significant effect on the magnitude of this disutility, indicating that respondents understand the framing that products only protect against HIV. Likewise, in both models, providing anal sex causes significant disutility in current practice, and the extent of this does not appear to change with the introduction of PrEP. There is no significant difference in preferences for any attribute, nor the impact of PrEP, among FSWs using contraception and those who are not, indicating that the impact of PrEP framing does not vary by pregnancy risk (results available from authors upon request).

The MMNL displays lower log‐likelihood, Akaike information criteria and Bayesian information criteria values than the MNL; however, these cannot be directly compared to assess model performance as the randomness introduced by the MMNL will inevitably affect these measures. However, the general consistency across MNL and MMNL specifications is reassuring. The differences that do occur are likely to be as a result of heterogeneity in preferences among the sample, which is not incorporated by the MNL.

Results suggest that the price premium for unprotected sex will reduce by 73% under full HIV protection, as denoted by the significant condom use and price parameters when interacted with framing. When the significance of the change in the ratio of these two parameters is assessed by the Delta method, the change in price premium due to protection is also statistically significant (*p* = 0.046).

The MNL model shows some evidence that the overall quantity of sex provided would increase under PrEP use, as the opt‐out parameter decreases significantly under PrEP framing. The lack of statistically significant parameters in the unframed DCE willingness to accept ratios, presented in Table S1, may be due to the lack of precision in the price estimate, which, in fact, further highlights the relative importance of the price attribute under PrEP use.

We assess the theoretical validity of DCE results by comparing data from HIV‐negative and ‐positive respondents, displayed in Table S2 showing MNL results for the unframed DCE by HIV status. The implied condom differential from DCE data is, as predicted theoretically, larger among HIV‐negative FSWs (ZAR 11,315) than HIV‐positive FSWs (ZAR 4,224), though this difference is not statistically significant when assessed by the Delta method (*p* = 0.3) or by examination of the individual parameters in the interactions of Model (4).

### 4.3 Simulations

Because the condomless sex price premium we observe from revealed preference data without PrEP is ZAR 394 (ZAR 466–ZAR 72), we predict that the premium will fall to ZAR 107. All else equal, with this reduced condom differential, PrEP use will lead to an increase in condomless sex by a factor of 2.27, compared with before the introduction of PrEP. If the premium were to remain constant at ZAR 394, the change in the utility provided by condoms and price indicates that the quantity of condomless sex would increase by a factor of 3.2.

### 4.4 Heterogeneity

We explore observed heterogeneity in our data though specifying interaction effects with several respondent characteristics shown in Table 5. The most important finding here is that the impact of PrEP does not vary among FSW subgroups (Model 6C); however, these null findings may be due to the number of parameters estimated in this model relative to the sample size. Although the effect of PrEP is consistent across the sample, there is some heterogeneity in preferences for the DCE attributes. Married FSWs value act price significantly less than their unmarried peers across both framings, and there is indicative evidence that recent experience of IPV and knowing other FSWs who engage in condomless sex may increase the influence of price under PrEP introduction.

**Table 5:** Heterogeneity in preferences—Interaction effects

	Model 6A: No framing MNL		Model 6B: PrEP framed MNL		Model 6C: Interacted MNL	
	Coeff.	*SE*	Coeff.	*SE*	Coeff.	*SE*
Price	−0.0005	0.0007	−0.0011	0.0006	−0.0005	0.0007
Condom use						
No condom						
Condom	4.39***	0.29	3.80***	0.28	4.39	0.29
Type of sex						
Vaginal						
Anal	−2.92***	0.18	−3.05***	0.18	−2.92	0.18
Perceived client HIV risk						
Do not think client has HIV						
Think client has HIV	−0.49***	0.14	−0.28***	0.14	−0.49	0.14
Perceived client STI risk						
Do not think client has STI						
Think client has STI	−0.87***	0.17	−0.75***	0.15	−0.87	0.17
Opt‐out (no services to either)	1.43***	0.17	0.92***	0.14	1.43***	0.17
Framing interactions						
PrEP framing × price					−0.0006	0.0009
PrEP framing × condom use					−0.59*	0.40
PrEP framing × anal sex					−0.13	0.25
PrEP framing × client HIV					0.21**	0.20
PrEP framing × client STI					0.12	0.23
PrEP framing × opt‐out					−0.51	0.22
Respondent characteristic interactions						
Price * married	−0.003**	0.005	−0.01***	0.004	−0.003**	−0.67
Price * low income	0.000	0.001	0.001	0.001	−0.0002	−0.23
Price * experience of IPV in previous 12 months	0.001	0.001	0.002**	0.001	0.001	1.78
Price * know FSWs who engage in unprotected sex	0.001	0.001	0.002***	0.001	0.001	1.78
Condom * married	1.404*	1.246	0.87	1.07	1.40*	1.13
Condom * low income	0.341	0.274	0.13	0.28	0.34	1.24
Condom * experience of IPV in previous 12 months	−0.070	0.292	0.37	0.31	−0.07	−0.24
Condom * know FSWs who engage in unprotected sex	−0.010	0.284	−0.55	0.29	−0.01	−0.03
Framing * respondent characteristic interactions						
Price * married * PrEP framing					−0.002	−0.35
Price * low income * PrEP framing					0.001	1.21
Price * experience of IPV in previous 12 months * PrEP framing					0.001	0.76
Price * know FSWs who engage in unprotected sex * PrEP framing					0.001	1.35
Condom * married * PrEP framing					−0.53	−0.32
Condom * low income * PrEP framing					−0.21	−0.54
Condom * experience of IPV in previous 12 months * PrEP framing					0.44	1.04
Condom * know FSWs who engage in unprotected sex * PrEP framing					−0.54	−1.34
Model diagnostics						
Log likelihood	−581.41		−671.39		−1252.79	
AIC	1,190.81		1,370.78		2,561.59	
BIC	1,260.70		1,440.78		2,720.95	
*N*	122		122		122	

*Note*. MNL: multinomial logit; PrEP: pre‐exposure prophylaxis; FSWs: female sex workers; AIC: Akaike information criteria; BIC: Bayesian information criteria.

*** Significant at 1%

** significant at 5%

* significant at 10%.

## 5 DISCUSSION

This study is the first to quantitatively consider the impact of PrEP on the economics of sex work. We use stated preference methods to estimate the effect of introducing effective HIV prevention products on the supply of commercial sex. HIV‐negative FSW participants completed two identical DCEs at the beginning and end of a larger survey, where the first DCE was framed in the context of current practice (i.e., without PrEP) and the later DCE framed as if FSWs were using a 100% effective HIV prevention product (i.e., with PrEP). Consistent with prior expectations, we found that PrEP use increased the influence of price on FSW choices whereas condoms were valued significantly less. Results strongly suggest that PrEP use could reduce the condom differential (price premium for condomless sex) and increase condomless commercial sex. There was some evidence of preference heterogeneity between married and unmarried FSWs; however, our results do not suggest that the response to PrEP introduction will vary much across FSWs. We find that the price of acts does not play an important part in FSW decisions at present but is likely to significantly impact choices after the introduction of PrEP. Our simulations of choice data indicate that the condom differential will reduce by 73% under PrEP use. Moreover, the quantity of condomless sex provided by FSWs is predicted to more than double, increasing by a factor of 2.27 under PrEP use.

There are three key reasons why this is an important consideration in the PrEP response. It is possible that if condom use falls after introducing PrEP, but FSWs are able use PrEP effectively, then the projected increase in condomless sex will not substantively affect the HIV epidemic. However, higher levels of condomless sex could increase the transmission of STIs, which may increase the risk of HIV transmission, thus dampening the benefits of PrEP (Freeman et al., 2007)—though theoretical modeling work has shown that this may not be important (Grant et al., 2017). Additionally, if PrEP users are unable to fully adhere to regimens, as observed in some trials, then the impact of PrEP may be markedly reduced. To date, there is mixed evidence of condom substitution from PrEP trials and demonstration projects. Some data from oral PrEP trials, largely among MSM populations, indicate that self‐reported condom use has not changed among PrEP users (Carlo Hojilla et al., 2016; Liu et al., 2013; Molina et al., 2015; Thomas et al., 2016). In contrast, a number of MSM studies have detected increased STI rates among PrEP users than nonusers, an objective indication of increased levels of condomless sex (de Wit et al., 2015; Lal et al., n.d.; Marcus et al., 2016; Montano et al., 2017). Mathematical modeling work suggests that, if present, condom substitution may substantively reduce the impact of partially effective products (Foss, Vickerman, Heise, & Watts, 2003; Punyacharoensin et al., 2016). Future work is required in this area.

This study suggests that PrEP could exacerbate the difficulties that FSWs face in negotiating condom use with commercial sexual partners, if clients are aware that FSWs are using PrEP. In this instance, where FSWs are economically vulnerable, or do not have sufficient negotiating power with clients to insist on condom use, PrEP may increase the occurrence of condomless sex. We also show that FSWs who increase the supply of unprotected sex with PrEP use are simply reacting rationally to exogenous market changes imposed by PrEP programs and should not face blame or stigma as a result. Instead, the unintended consequences of PrEP introduction should be fully considered by program implementers through collection of reliable data on act price and condom use, alongside coercion from clients to provide condomless sex.

This paper is the first to estimate the effect of new HIV prevention products on the supply of condomless commercial sex. These findings are important for HIV prevention programs among FSWs as they suggest that condom use may fall after the introduction of effective PrEP products, in part due to the increased importance of act price. Although expected by some (Blumenthal & Haubrich, 2014), self‐reported condom use among PrEP users has not fallen substantially in trials among MSM, FSWs, or general populations (Guest et al., 2008; Molina et al., 2015). Yet, self‐reported condom use data could be substantially overreported, for example, a list randomization study among FSWs in Senegal showed that condom use was overestimated by 20% points.

There are strong scientific, economic, and human rights arguments for implementing PrEP among FSWs as soon as possible (Bekker et al., 2015), and rollout has begun in many countries including South Africa (Department of Health South Africa, 2016). The findings of this study do not reduce the public health imperative to make effective HIV prevention products available to FSWs, rather we suggest three things: (a) the unintended consequences of PrEP implementation should be explicitly measured to assess how the incentive structure of sex work will change as a result of PrEP introduction; (b) FSWs who may struggle to adhere consistently to PrEP should be identified and supported in their adherence, particularly if they are also women who are less able to use condoms consistently; and (c) the impact of PrEP provision on client attitudes and demands for protected and condomless sex should be monitored, as clients (who already have a strong bargaining position) may place excessive pressure on FSWs perceived to be using PrEP to provide condomless sex.

Importantly, these results indicate that a degree of substitution from condom‐ to PrEP‐protected sex is a rational response to the use of an effective HIV prevention product, and more work is needed to explore how public health programs can best support FSWs to protect their own sexual and reproductive health. A promising option is the introduction of multipurpose HIV prevention products, co‐formulated or co‐packaged combinations of HIV, STI, and/or contraceptive compounds, which have been estimated to be cost‐effective among FSWs in South Africa (Quaife et al., 2018). In addition, PrEP use may increase FSW contact with the health system as women collect monthly or three‐monthly prescriptions (Eakle et al., 2017). The South African national guidelines for PrEP implementation indicate that syndromic STI screening will be carried out at each visit, alongside HIV testing and behavioral sexual risk reduction counseling, whereas a urine pregnancy test will be carried out at PrEP initiation (Department of Health South Africa, 2016). This increased contact with the health system may increase overall FSW health in addition to direct benefits from HIV prevention. For example, in North America, patients receiving ARV therapy have an increased likelihood of being screened for cardiovascular disease, hepatitis C infection, and cancer (Trickey et al., 2017).

This paper has several limitations. First, we use stated preference data on FSW choices because observational revealed preference data from PrEP trials or programs are not yet available, and results may be affected by hypothetical bias, yet the direction in which this bias might impact results is not clear. The small amount of information on which FSWs make decisions in this study compared with the real world means that the impact of the framing may be overstated, and we therefore overpredict behavioral changes. By contrast, the salience of PrEP use in reality (e.g., by taking one tablet every day) may increase the perception of protection to a greater extent than the framing of a choice task, making these results conservative. Reassuringly, however, evidence from health‐focused studies suggests that stated and revealed preferences may be closely correlated (Quaife, Terris‐Prestholt, di Tanna, & Vickerman, 2018; Telser & Zweifel, 2007). Furthermore, at the cost of potential hypothetical bias, we are able to avoid endogeneity present in observational studies, as some (e.g., risk averse) FSWs may be both more likely to opt‐in to PrEP use and to only provide protected sex. We note that although we considered ways to make the hypothetical tasks more realistic, for example, by having real photos or profiles of clients as choice tasks, or when FSWs chose a client in reality showing them a hypothetical alternative, these were not practically or ethically possible in the busy, largely brothel‐based data collection process. During the survey, participants were given physical examples of potential prevention products to imagine real‐world use.

Second, the choice task itself assumes that FSWs have a substantial degree of agency over their choice of clients, which may not be accurate. However, qualitative research during the design of this study suggests that the large pool of potential clients in this context makes this a reasonable assumption (Eakle et al., 2016), whereas the RDS sample necessarily identified FSWs with some link to peer‐educators who are likely to have higher self‐efficacy than FSWs not reached by clinical or peer networks. This assumption may not hold for other sex work contexts in South Africa, or beyond. Third, the DCE is a simplistic representation of choices in sex work. The description of PrEP use, describing a perfectly effective product with no adherence requirements, does not reflect the imperfect effectiveness of PrEP. However, this was a pragmatic decision to simplify after extensive piloting showed that participants found it difficult to understand frames describing imperfect effectiveness or additional protection from STIs. We acknowledge that this may overstate that behavioral response to HIV protection and that this is a limitation of this study. An improved study design would have shown scenarios with varying levels of efficacy and assessed if condom substitution had a dose–response relationship with the amount of protection provided; future work should consider the implications of this on sample size and participant fatigue. It is also unrealistic to assume that FSWs can reliably judge client STI or HIV status.

Fourth, HIV status was assessed through participant self‐report not objective HIV testing, which was not feasible as part of this survey. If present, acceptability bias would likely lead to underreporting of HIV‐positive status, though it is not clear how including HIV‐positive FSWs in this analysis would affect results. Indeed, HIV‐positive respondents may understate a behavioral response to use a prevention product, with condoms chosen more often due to the salient, negative consequences of HIV infection. Furthermore, FSWs may not know their HIV‐positive status through a lack, or avoidance, of testing. Because this study implicitly asked respondents to make choices according to their perceived HIV status, this study estimates the behavioral response to prevention products of FSWs who perceive themselves to be HIV negative, although in reality they may not be. Recent work from South Africa found that self‐reported HIV status had a high positive predictive value (94%) but a low negative predictive value (87%) compared with a biomarker test; this underestimate in positive status was largely explained by time since last test, suggesting that self‐reported HIV status correlates well with perceived HIV status.

Fifth, we do not consider the demand side of the commercial sex market, specifically the impact of PrEP use among FSWs on the preferences of clients. If clients know, or perceive FSWs to be using PrEP, they could use the reduced risk of HIV acquisition in coercive arguments for unprotected sex. Finally, the diverse nature of sex work within South Africa and across sub‐Saharan Africa makes generalizability difficult to assess from this small sample of FSWs.

## 6 CONCLUSION

We explored how the introduction of new HIV prevention products might influence the economics and supply of condomless commercial sex. By applying stated preference methods in the form of a repeated DCE, we show that introducing a 100% effective HIV prevention product may double the number of condomless acts supplied and considerably reduce (by 73%) the price premium for condomless sex through decreasing the value placed on condoms and increasing the utility obtained from act price. These findings have implications for the possible impact of PrEP rollout among FSW groups, which is proceeding worldwide, especially in scenarios where women find it hard to effectively adhere to PrEP. In these settings, the reduced use of condoms may reduce the impact of PrEP. Further research is needed on the revealed preferences of FSWs and clients to assess how PrEP and associated economic incentives related to condomless sex affect health choices in commercial sex to allow appropriate support interventions to be put in place.

## CONFLICTS OF INTEREST

None to declare

## AUTHOR CONTRIBUTIONS

M. Q., P. V., and F. T. P. conceptualized the study. M. C., R. E., and S. D. M. advised on study design. M. Q. wrote the first draft. S. M. wrote the theoretical model. M. Q., S. M., P. V., R. E., M. C., S. D. M., and F. T. P. critically reviewed the drafts and approved the final version.

## Supporting information


**Table S1:** Willingness to accept ratios
**Supplementary table S2:** Comparison of preferences by HIV status
