# Supplementary material for: The effect of HIV prevention products on incentives to supply condomless commercial sex among female sex workers in South Africa
Source: Health Econ. 2018 Jun 21;27(10):1550–66. doi: 10.1002/hec.3784 (PMC6175015; doi:10.1002/hec.3784)
Supplement: Supplementary file 1 — Table S1: Willingness to accept ratios Supplementary table S2: Comparison of preferences by HIV status [file HEC-27-1550-s001.docx]

## Supplementary table S1: Willingness to accept ratios

|  |  | WTA: No framing MMNL | | | WTA: PrEP framed MMNL | | |
| --- | --- | --- | --- | --- | --- | --- | --- |
|  |  | Coeff. |  | SE | Coeff. |  | SE |
| **Price** | | 1 | - | - | 1 | - | - |
| **Condom use** | |  |  |  |  |  |  |
|  | Condom*PrEP framing | 11721.8 |  | 10436.5 | 3184.3 | *** | 804.9 |
| **Type of sex** | |  |  |  |  |  |  |
|  | Anal*PrEP framing | -7529.0 |  | 6907.2 | -2572.1 | *** | 709.4 |
| **Perceived client HIV risk** | |  |  |  |  |  |  |
|  | Think client has HIV*PrEP framing | -1240.4 |  | 1117.4 | -207.3 | ** | 87.2 |
| **Perceived client STI risk** | |  |  |  |  |  |  |
|  | Think client has STI*PrEP framing | -2156.5 |  | 2150.7 | -549.3 | *** | 174.1 |
| **Opt-out (no services to either)** | |  |  |  |  |  |  |
|  | Opt-out*PrEP framing | 3795.5 |  | 3135.1 | 860.6 | *** | 227.1 |

## Supplementary table S2: Comparison of preferences by HIV status

|  |  |  | **(1)** |  |  | **(2)** |  |  | **(3)** |  |  | **(4)** |  |
| --- | --- | --- | --- | --- | --- | --- | --- | --- | --- | --- | --- | --- | --- |
|  |  | **Whole sample** | | | **HIV-positive** | | | **HIV-negative** | | | **Interaction model** | | |
|  |  | **Coeff.** |  | **SE** | **Coeff.** |  | **SE** | **Coeff.** |  | **SE** | **Coeff.** |  | **SE** |
| **Price** |  | 0.0006 | *** | 0.0003 | 0.0008 | ** | 0.0004 | 0.0004 |  | 0.0005 | 0.0009 | *** | 0.0003 |
| **Condom use** | |  |  |  |  |  |  |  |  |  |  |  |  |
|  | No condom |  |  |  |  |  |  |  |  |  |  |  |  |
|  | Condom | 3.9543 | *** | 0.1658 | 3.3788 | *** | 0.2314 | 4.5258 | *** | 0.2434 | 4.0703 | *** | 0.1642 |
| **Type of sex** | |  |  |  |  |  |  |  |  |  |  |  |  |
|  | Vaginal |  |  |  |  |  |  |  |  |  |  |  |  |
|  | Anal | -2.5147 |  | 0.1227 | -2.1307 |  | 0.1708 | -2.8913 |  | 0.1776 | -2.9205 |  | 0.1233 |
| **Perceived client HIV risk** | |  |  |  |  |  |  |  |  |  |  |  |  |
|  | Do not think client has HIV |  |  |  |  |  |  |  |  |  |  |  |  |
|  | Think client has HIV | -0.556 |  | 0.1023 | -0.652 |  | 0.1486 | -0.4763 |  | 0.1428 | -0.339 |  | 0.0969 |
| **Perceived client STI risk** | |  |  |  |  |  |  |  |  |  |  |  |  |
|  | Do not think client has STI |  |  |  |  |  |  |  |  |  |  |  |  |
|  | Think client has STI | -0.6011 |  | 0.1094 | -0.4142 |  | 0.149 | -0.8281 |  | 0.1639 | -0.7116 |  | 0.1081 |
| **Opt-out (no services to either)** | | 1.1347 | *** | 0.1148 | 0.7174 | *** | 0.1633 | 1.4575 | *** | 0.1659 | 1.2283 | *** | 0.1053 |
| **Interactions** | |  |  |  |  |  |  |  |  |  |  |  |  |
|  | HIV positive x price |  |  |  |  |  |  |  |  |  | -0.0001 |  | 0.0005 |
|  | HIV positive x condom use |  |  |  |  |  |  |  |  |  | -0.6916 |  | 0.2838 |
|  | HIV positive x anal sex |  |  |  |  |  |  |  |  |  | 0.7898 | ** | 0.2106 |
|  | HIV positive x client HIV |  |  |  |  |  |  |  |  |  | -0.3129 |  | 0.1774 |
|  | HIV positive x client STI |  |  |  |  |  |  |  |  |  | 0.2975 | * | 0.1841 |
|  | HIV positive x opt-out |  |  |  |  |  |  |  |  |  | -0.511 |  | 0.1943 |
|  |  |  |  |  |  |  |  |  |  |  |  |  |  |
| **Implied condom differential (ZAR)** | | 6,591 |  |  | 4,224 |  |  | 11,315 |  |  |  |  |  |
